# Effect of weight loss on circulating fatty acid profiles in overweight subjects with high visceral fat area: a 12-week randomized controlled trial

**DOI:** 10.1186/s12937-018-0323-4

**Published:** 2018-02-22

**Authors:** Young Ju Lee, Ayoung Lee, Hye Jin Yoo, Minjoo Kim, Minkyung Kim, Sun Ha Jee, Dong Yeob Shin, Jong Ho Lee

**Affiliations:** 1 National Leading Research Laboratory of Clinical Nutrigenetics/Nutrigenomics, Department of Food and Nutrition, College of Human Ecology, Yonsei University, 50 Yonsei-ro, Seodaemun-gu, Seoul, 03722 Korea; 2 Department of Food and Nutrition, Brain Korea 21 PLUS Project, College of Human Ecology, Yonsei University, 50 Yonsei-ro, Seodaemun-gu, Seoul, 03722 Korea; 3 Research Center for Silver Science, Institute of Symbiotic Life-TECH, Yonsei University, 50 Yonsei-ro, Seodaemun-gu, Seoul, 03722 Korea; 4 Department of Epidemiology and Health Promotion, Institute for Health Promotion, Graduate School of Public Health, Yonsei University, 50 Yonsei-ro, Seodaemun-gu, Seoul, 03722 Korea; 5 Department of Internal Medicine, Severance Hospital, Division of Endocrinology and Metabolism, Yonsei University College of Medicine, 50-1 Yonsei-ro, Seodaemun-gu, Seoul, 03722 Korea

**Keywords:** Fatty acid, Fatty acid desaturase, Visceral fat, Weight loss, Obesity-related disease

## Abstract

**Background:**

Significant associations between visceral fat and alterations in plasma fatty acids have been identified in overweight individuals. However, there are scant data regarding the relationships of the visceral fat area (VFA) with the plasma fatty acid profiles and desaturase activities following weight loss. We investigated the effect of weight loss with mild calorie restriction on the circulating fatty acid profiles and desaturase activities in nondiabetic overweight subjects with high VFA.

**Methods:**

Eighty overweight subjects with high VFA (L4 VFA ≥100 cm^2^) were randomized into the 12-week mild-calorie-restriction (300 kcal/day) or control groups.

**Results:**

Comparison of the percent of body weight changes between groups revealed that the weight-loss group had greater reductions in body weight. The VFA decreased by 17.7 cm^2^ from baseline in the weight-loss group (*P* < 0.001). At follow-up, the weight-loss group showed greater reductions in serum triglycerides, insulin, and HOMA-IR than the control group. Significantly greater reductions in total saturated fatty acids, palmitic acid, stearic acid, total monounsaturated fatty acids, palmitoleic acid, oleic acid, eicosadienoic acid, and dihomo-γ-linolenic acid levels were detected in the weight-loss group compared with the control group after adjusting for baseline values. Following weight loss, C16 Δ9-desaturase activity was significantly decreased and Δ5-desaturase activity was significantly increased, and the changes were greater in the weight-loss group than in the control group.

**Conclusions:**

The results suggest that mild weight loss improves abdominal obesity, overall fatty acid profiles, and desaturase activities; therefore, mild calorie restriction has potential health benefits related to obesity-related diseases in overweight subjects with high VFA.

**Trial registration:**

NCT02992639. Retrospectively registered 11 December 2016.

## Background

A visceral fat area (VFA) cutoff of 100 cm^2^ was established to predict the risk of obesity-related health risks, including insulin resistance (IR), metabolic syndrome, and diabetes, in Asian populations [1]. Compared with abdominal subcutaneous fat, visceral fat is more metabolically active in the fasting state [2, 3]. Compared with other weight-loss methods, calorie restriction tends to reduce more visceral than subcutaneous adipose tissue [4]. It is known that plasma fatty acid composition mirrors not only dietary fatty acid composition but also endogenous fatty acid synthesis, in which desaturases play important roles [5]. In endogenous fatty acid synthesis, C16 Δ9-desaturase (C16:1/C16:0) activity is known to be high in conditions of diabetes and abdominal obesity. Additionally, an inverse relation between Δ5-desaturase activity (C20:4, n-6/C20:3, n-6) and diabetes risk and a direct relation between Δ6-desaturase activity (C18:3, n-6/C18:2, n-6) and IR have been reported. Pan et al. [6] reported that obesity is related to increased Δ9-desaturase activity and reduced Δ5-desaturase activity. Several previous studies observed that low Δ5-desaturase activity is linked to increased risk of type 2 diabetes and insulin resistance among Japanese adults [7, 8]. Zhao et al. [9] also noted that Δ5-desaturase activity is inversely associated with metabolic abnormalities among obese Chinese subjects. In a Swedish study, Δ5-desaturase activity was found to be inversely associated with obesity and insulin resistance, whereas Δ6-desaturase activity demonstrated positive associations [10].

Significant associations between visceral fat amount and alterations in serum fatty acids have been identified in overweight individuals [11]. However, there are scant data regarding the relations between changes in VFA and changes in circulating fatty acid profiles and desaturase activities following weight loss. Research is required to determine the amount of visceral fat reduction with calorie restriction that is necessary to induce favorable metabolic changes in the overall fatty acid profile. Therefore, in this study, we investigated the effects of weight loss with mild calorie restriction (a 300 kcal/day intake reduction) on circulating fatty acid profiles and desaturase activities in nondiabetic overweight subjects with high VFA [VFA at the 4th lumbar vertebrae (L4 VFA) ≥ 100 cm^2^].

## Methods

### Study subjects

Study subjects were recruited through advertisements posted in Seoul. Based on the data screened by the Clinical Nutrition Lab, Yonsei University, overweight subjects [25.0 kg/m^2^ ≤ body mass index (BMI) < 30 kg/m^2^] were referred to the Department of Internal Medicine, Yonsei University Severance Hospital. After their health and basic blood tests, including serum glucose, were rechecked, individuals who met the study criteria were recommended for study participation. The inclusion criteria were as follows: age between 20 and 60 years; absence of pregnancy or breastfeeding; stable body weight (body weight change < 1 kg in the 3 months before screening); BMI between 25 and 30 kg/m^2^; high L4 VFA (L4 VFA ≥ 100 cm^2^); absence of hypertension, type 2 diabetes, cardiovascular disease, and thyroid disease; and no use of medication affecting body weight, energy expenditure, or glucose control for 6 months prior to screening. Subjects were excluded if they had a history of Cushing syndrome, malignancy, or liver disease, including chronic viral hepatitis, autoimmune hepatitis, primary biliary cirrhosis, and drug-induced liver disease. Male and female subjects whose alcohol consumption was > 40 and > 20 g/day, respectively, and subjects with a history of intentional weight reduction in the 6 months before the current study were excluded. Finally, those who consented to participate in the program were included in this study. The purpose of the study was carefully explained to all participants, and written consent was obtained prior to their participation. The protocol was approved by the Institutional Review Board of Yonsei University and Yonsei University Severance Hospital in accordance with the Helsinki Declaration.

### Study design and intervention

The basic framework of the present study was based on a previous study [12]. A 12-week, placebo-controlled, randomized study was conducted with 80 high VFA, nondiabetic overweight subjects (L4 VFA ≥ 100 cm^2^). Subjects were divided into two groups: a weight-loss group with 12 weeks of mild calorie restriction (a 300 kcal/day intake reduction) or a control group with no treatment (NCT02992639; http://www.clinicaltrials.gov/). Randomization was achieved by computer-generated block randomization (placebo:test = 1:1).

### Protocol for weight loss

The subjects in the weight-loss group followed a 12-week weight-loss program comprising a 300 kcal/day reduction of their usual caloric intake. Basically, the subjects were instructed to remove 1/3 of a bowl of rice (approximately 100 kcal; a bowl of rice is approximately 300 kcal per meal, according to the food composition table from the Rural Development Administration of Korea [13]) from one meal a day for easier application of the 300 kcal/day deficit. Moreover, based on each subject’s reported food intake, an individualized and nutritionally balanced diet plan was produced by a trained nutritionist to achieve the goal of losing a minimum of 3% of initial body weight. The instructions included food choices, cooking methods, reductions in snack consumption frequency, low-calorie substitutions for high-calorie foods, low-fat foods, and limitations on simple sugar consumption. The subjects in the control group were instructed to maintain their usual dietary and physical activity habits during the study period.

### Daily energy intake and physical activity measurements

At baseline, the subjects’ usual dietary intakes were assessed via 24-h recall and semiquantitative food frequency questionnaires completed with the nutritionist’s assistance. In addition, a standardized 3-day dietary record was obtained from each participant at week 6 and 12. These records were completed at home following detailed instructions from the nutritionist. A computerized version of the Korean Nutrition File (Can-Pro 3.0; The Korean Nutrition Society, Seoul, Korea) was used to determine the macronutrient contents of foods and total daily energy intakes. On the same days as the dietary records, 3-day physical activity records were completed at home. The total energy expenditure (TEE) (kcal/day) was calculated from activity patterns, including the basal metabolic rate (BMR), physical activity for 24 h, and the specific dynamic action of food. Each subject’s BMR was calculated using the Harris-Benedict equation.

### Anthropometric parameters and body composition measurements

Detailed information was previously published [14]. Briefly, all data were acquired at baseline and week 12. Body weight (Inbody370; Biospace, Cheonan, Korea), height, waist circumference, and blood pressure (BP) were measured. BMI was calculated in units of kilogram per square meter (kg/m^2^). The abdominal fat distribution was measured at L4 by computed tomography (CT). The scanning parameters were a slice thickness of 1 mm at 200 mA and 120 kVp, with a 48-cm field of view. Abdominal adipose tissue was determined using the attenuation range from − 150 to − 50 Hounsfield units in the CT images. The body composition of the study participants was measured via dual-energy X-ray absorptiometry (DEXA) to determine the fat percentage, fat mass, and lean body mass.

### Blood collection and biochemical assessments

Details were provided previously [14]. Blood samples were collected after an overnight fast of at least 12 h. Venous blood specimens were collected in EDTA-treated tubes and serum tubes. The blood samples were centrifuged to obtain plasma and serum samples, which were then stored at − 70 °C. The levels of serum-fasting glucose, insulin, triglycerides (TGs), total cholesterol, and high-density lipoprotein (HDL) cholesterol were measured [15]. The IR and low-density lipoprotein (LDL) cholesterol levels were calculated using the equations of homeostatic model assessment (HOMA) and the Friedewald formula, respectively: HOMA-IR = [fasting insulin (μIU/mL) × fasting glucose (mg/dL)]/405; Friedewald formula: LDL cholesterol = total cholesterol – [HDL cholesterol + (TG/5)].

### Metabolite extraction and gas chromatography–mass spectrometry (GC-MS) analysis

The details of the GC-MS procedure have been previously published [16]. Briefly, plasma samples (100 μL) were placed into PTEE screw-capped Pyrex tubes. An internal standard compound containing heptadecanoic acid (500 μL, 25 ppm in n-hexane) and methanol (2 mL) were added to the samples. After vortexing, acetyl chloride (100 μL) was added slowly, and the tubes were then heated at 95 °C for 1 h. After cooling on ice, 6% potassium carbonate (5 mL) was added to the tubes. The tubes were centrifuged (4 °C, 3000 rpm, 5 min), and the clear n-hexane top layer (100 μL), including the fatty acid methyl esters (FAME), was then transferred into a vial prior to GC-MS analysis.

GC-MS analyses were performed on an Agilent Technologies 7890 N gas chromatograph coupled with an Agilent Technologies 5977A quadrupole mass selective spectrometer with a triple-axis detector (Agilent, Palo Alto, CA, USA) in the electron ionization (70 eV) and full scan monitoring (m/z 50–800) modes. The injection volume of the samples was 1 μL in the splitless mode. The fatty acid methyl esters of all samples were separated on a VF-WAX column (Agilent Technologies, Middelburg, Netherlands) with helium as a carrier gas. The temperature program was as follows: (1) initial temperature of 50 °C for 2.3 min, (2) increase to 175 °C at 50 °C/min, and (3) increase to 230 °C at 2 °C/min. The metabolites in the samples were identified by comparing their relative retention times and mass spectra with those of authentic reference standards. The relative levels of metabolites were calculated by comparing their peak areas to those of the internal standard compound.

### Statistical analysis

The statistical analyses were conducted with SPSS version 23.0 software (IBM/SPSS, Chicago, IL, USA). Skewed variables were logarithmically transformed. An independent *t*-test compared continuous variables between the control and weight-loss groups. A paired *t*-test was used to compare continuous variables at baseline and follow-up within each group. For the comparison of categorical variables, a chi-squared test was conducted. A general linear model test was performed to adjust the baseline values of the changed values. A Pearson’s correlation coefficient was used to examine the relations among variables. A heat map was created using Multiple Experiment Viewer (MeV) version 4.9.0 (http://www.tm4.org/) to visualize and evaluate the relations among variables in the study population. The results are expressed as the mean ± standard error (SE). A two-tailed *P* < 0.05 was considered statistically significant.

## Results

The dropout rate was less than 10% in the control (*n* = 2) and weight-loss (*n* = 3) groups. Participants were excluded for personal reasons (*n* = 4) and lack of a blood sample for GC-MS analysis (*n* = 1).

### Effects of mild calorie restriction on clinical and biochemical characteristics at baseline and follow-up

The age, sex distribution, smoking, and drinking did not significantly differ between the two groups (Table 1). At baseline, no significant differences were observed in all clinical and biochemical parameters between the control and weight-loss groups; after the 12-week intervention, the BMI, serum insulin levels, HOMA-IR score, and serum TG levels were significantly decreased in the weight-loss group compared with the control group (Table 1). In the control group, the subjects showed slight but significant weight gain (weight and BMI; all *P* < 0.001) during the intervention, whereas the subjects in the weight-loss group showed significant reductions in not only the weight and BMI (both *P* < 0.001) but also the waist circumference (*P* < 0.001), systolic BP (*P* = 0.049), insulin levels (*P* = 0.001), and HOMA-IR score (*P* = 0.003) following the intervention (Table 1). In addition, these reductions in weight, BMI, and waist circumference in the weight-loss group were greater than those in the control group after adjusting for the baseline values (all *P* < 0.001) (Table 1). The serum insulin levels, HOMA-IR score, and serum TG levels also showed significantly greater decreases in the weight-loss group than in the control group after adjusting for the baseline values (*P* = 0.020, *P* = 0.034, and *P* = 0.022, respectively) (Table 1). The estimated TEE (2144.6 ± 45.3 kcal/d vs. 2201.3 ± 59.3 kcal/d; *P* = 0.519), total calorie intake (TCI) (2105.8 ± 40.0 kcal/d vs. 2200.5 ± 50.3 kcal/d; *P* = 0.159), and percentage of macronutrient intakes (%CHO:%PRO:%FAT = 61:15:22 in both groups) did not significantly differ between the two groups at baseline (data not shown). The subjects’ overall compliance with the weight-loss plan was good. The estimated TCI of the weight-loss group at 12-week follow-up was, on average, 1949.4 kcal (%CHO:%PRO:%FAT = 59:19:22); and the types of dietary fats [saturated fatty acids (SFAs), monounsaturated fatty acids (MUFAs), and polyunsaturated fatty acids (PUFAs)] ingested by the study participants did not demonstrate any changes during the intervention (data not shown).

**Table 1:** Clinical and biochemical characteristics of the weight-maintenance (control) and weight-loss groups at baseline and 12-week follow-up

	Weight-maintenance group (*n* = 38)			Weight-loss group (*n* = 37)			*P* ^*a*^	*P* ^*b*^	*P* ^*c*^	*P* ^*d*^
	Baseline		Follow-up	Baseline		Follow-up				
Age (year)		46.0 ± 1.35			44.1 ± 1.92		0.421			
Male/female *n*, (%)		11 (28.9)/27 (71.1)			15 (40.5)/22 (59.5)		0.292			
Cigarette smoker *n*, (%)		4 (10.5)			5 (13.5)		0.691			
Drinker of alcohol *n*, (%)		21 (55.3)			22 (59.5)		0.713			
Weight (kg)	71.6 ± 1.51		72.1 ± 1.49^***^	73.8 ± 1.54		71.4 ± 1.52^***^	0.321	0.743		
Change		0.44 ± 0.10			− 2.42 ± 0.24				< 0.001	< 0.001
BMI (kg/m^2^)	27.3 ± 0.25		27.4 ± 0.24^***^	27.3 ± 0.23		26.4 ± 0.24^***^	0.994	0.002		
Change		0.17 ± 0.04			− 0.90 ± 0.08				< 0.001	< 0.001
Waist (cm)	93.6 ± 0.82		93.4 ± 0.83	93.0 ± 0.86		91.5 ± 0.80^***^	0.648	0.103		
Change		− 0.13 ± 0.21			− 1.48 ± 0.31				0.001	< 0.001
Systolic BP (mmHg)	122.4 ± 1.99		123.0 ± 2.49	126.1 ± 2.20		122.9 ± 2.09^*^	0.214	0.431		
Change		− 2.09 ± 1.87			− 3.23 ± 1.59				0.645	0.919
Diastolic BP (mmHg)	77.6 ± 1.67		76.9 ± 1.60	78.0 ± 1.52		76.7 ± 1.47	0.871	0.930		
Change		− 0.70 ± 1.26			− 1.26 ± 1.30				0.758	0.794
Glucose (mg/dL)^*∮*^	89.5 ± 1.46		90.8 ± 1.45	89.5 ± 1.65		89.9 ± 1.45	0.940	0.638		
Change		1.32 ± 1.40			0.41 ± 1.44				0.651	0.303
Insulin (μIU/dL)^*∮*^	15.5 ± 1.19		17.5 ± 2.25	13.5 ± 0.92		11.0 ± 0.75^**^	0.240	< 0.001		
Change		1.99 ± 1.91			− 2.61 ± 0.81				0.031	0.020
HOMA-IR^*∮*^	3.43 ± 0.26		4.00 ± 0.59	2.99 ± 0.20		2.42 ± 0.17^**^	0.256	< 0.001		
Change		0.57 ± 0.52			− 0.57 ± 0.19				0.046	0.034
Triglycerides (mg/dL)^*∮*^	166.4 ± 18.4		159.6 ± 12.9	135.9 ± 9.38		115.3 ± 7.56	0.271	0.010		
Change		− 6.74 ± 14.1			− 16.3 ± 8.36				0.565	0.022
Total-cholesterol (mg/dL)^*∮*^	220.30 ± 7.10		220.0 ± 6.93	213.8 ± 5.46		216.0 ± 6.18	0.577	0.738		
Change		− 0.37 ± 4.22			2.22 ± 4.90				0.690	0.857
HDL-cholesterol (mg/dL)^*∮*^	51.0 ± 1.97		51.6 ± 1.86	52.0 ± 1.60		54.0 ± 1.92	0.568	0.409		
Change		0.53 ± 1.34			2.00 ± 1.27				0.427	0.352
LDL-cholesterol (mg/dL)^*∮*^	136.0 ± 6.00		136.5 ± 6.31	134.6 ± 4.78		135.9 ± 4.90	0.911	0.855		
Change		0.45 ± 3.51			1.10 ± 3.86				0.901	0.933

Mean ± SE. ^∮^tested following logarithmic transformation. *P*^*a*^-values derived from an independent *t-*test between the groups at baseline. *P*^*b*^-values derived from an independent *t-*test between the groups at follow-up. *P*^*c*^-values derived from an independent *t-*test between the groups at changed values. *P*^*d*^-values are *P*^*c*^-values adjusted with the baseline values. ^*^P < 0.05, ^**^P < 0.01, and ^***^P < 0.001 derived from a paired *t*-test

### Effects of a 12-week mild calorie restriction on percent of body weight change, abdominal fat areas (CT), and body composition (DEXA)

Table 2 presents the effects of a 12-week mild calorie-restricted diet on the percent of body weight change, abdominal fat area measured by CT, and body composition measured by DEXA. Comparison of the percent of body weight changes (differences from baseline) revealed that the weight-loss group had greater reductions in the percent of body weight change than the control group (0.63 ± 0.15 vs. -3.44 ± 0.34; *P* < 0.001). Regarding the results of CT, no significant differences were observed in the L4 abdominal fat area (whole fat area and VFA), L4 subcutaneous fat area, or visceral-to-subcutaneous area ratio (VSR) at baseline between the control and weight-loss groups. At follow-up, the L4 VFA in the weight-loss group was lower than in the control group (*P* < 0.001). The VFA decreased by 17.7 cm^2^ (15%) from baseline in the weight-loss group (*P* < 0.001), and the VSR decreased by 0.09 from baseline in the weight-loss group (*P* < 0.001). Significantly greater reductions in the L4 VFA and VSR were observed in the weight-loss group compared with the control group (all *P* < 0.001). No statistically significant shifts in the VFA, subcutaneous fat area, or VSR were identified in the control group (Table 2). With regard to the DEXA results, there were no significant differences in the fat percentage, fat mass, or lean body mass between the two groups at both baseline and follow-up (Table 2). After the intervention, significant decreases in the fat percentage and fat mass were observed in both the control and weight-loss groups; however, greater reductions in the fat percentage and fat mass were observed in the weight-loss group than in the control group after adjustment for the baseline values (*P* = 0.006 and *P* < 0.001, respectively). The control group showed a significant increase in the lean body mass, whereas the weight-loss group showed a significant decrease in the lean body mass at the 12-week follow-up; these changes were significantly different between the two groups (*P* < 0.001) (Table 2).

**Table 2:** CT and DEXA evaluation of the weight-maintenance (control) and weight-loss groups at baseline and 12-week follow-up

	Weight-maintenance group (*n* = 38)			Weight-loss group (*n* = 37)			*P* ^*a*^	*P* ^*b*^	*P* ^*c*^	*P* ^*d*^
	Baseline		Follow-up	Baseline		Follow-up				
Percent weight change		0.63 ± 0.15			− 3.44 ± 0.34		< 0.001			
CT evaluation (L4)										
Whole fat area (cm^2^)	327.0 ± 6.32		333.3 ± 6.23	319.8 ± 6.65		298.5 ± 7.23^***^	0.435	< 0.001		
Change		6.34 ± 4.72			− 21.3 ± 4.70				< 0.001	< 0.001
Visceral fat area (cm^2^)	118.2 ± 2.34		121.9 ± 3.26	118.3 ± 3.14		100.6 ± 3.67^***^	0.989	< 0.001		
Change		3.65 ± 2.31			− 17.7 ± 3.30				< 0.001	< 0.001
Subcutaneous fat area (cm^2^)	208.7 ± 6.98		211.4 ± 6.24	201.5 ± 7.06		197.8 ± 6.90	0.468	0.148		
Change		2.69 ± 4.17			− 3.66 ± 2.54				0.200	0.099
Visceral/subcutaneous fat ratio (%)	0.60 ± 0.03		0.60 ± 0.03	0.62 ± 0.03		0.53 ± 0.03^***^	0.556	0.101		
Change		0.00 ± 0.02			− 0.09 ± 0.02				< 0.001	< 0.001
DEXA evaluation										
Fat percentage (%)	31.5 ± 0.94		31.0 ± 0.94^**^	29.8 ± 1.03		28.5 ± 0.98^***^	0.209	0.067		
Change		− 0.50 ± 0.18			− 1.26 ± 0.24				0.014	0.006
Fat mass (g)	22,826.7 ± 616.6		22,536.71 ± 606.1^*^	22,038.8 ± 685.1		20,768.91 ± 651.1^***^	0.395	0.050		
Change		− 290.0 ± 128.4			− 1269.9 ± 221.4				< 0.001	< 0.001
Lean body mass (g)	48,289.8 ± 1471.4		48,934.6 ± 1497.4^***^	50,875.5 ± 1598.1		49,726.2 ± 1567.7^***^	0.237	0.716		
Change		644.8 ± 168.6			− 1149.3 ± 164.3				< 0.001	< 0.001

Mean ± SE. ^∮^tested following logarithmic transformation. *P*^*a*^-values derived from an independent *t-*test between the groups at baseline. *P*^*b*^-values derived from an independent *t-*test between the groups at follow-up. *P*^*c*^-values derived from an independent *t-*test between the groups at changed values. *P*^*d*^-values are *P*^*c*^-values adjusted with the baseline values. ^*^P < 0.05, ^**^P < 0.01, and ^***^P < 0.001 derived from a paired *t*-test

### Effects of mild calorie restriction on circulating fatty acid profiles and desaturase activities at baseline and follow-up

Table 3 presents the effects of a 12-week mild calorie-restricted diet on plasma fatty acid profiles. At baseline, no significant difference was observed in the SFAs, MUFAs, n-6 PUFAs, and n-3 PUFAs between the control and weight-loss groups. The total SFAs, including palmitic acid (C16:0) and stearic acid (C18:0), decreased from baseline in the weight-loss group; in addition, these reductions were significantly greater than those in the control group after adjusting for the baseline values (*P* = 0.027, *P* = 0.033, and *P* = 0.021, respectively). At follow-up, the weight-loss group showed significantly lower levels of total SFAs (*P* = 0.029), lauric acid (C12:0) (*P* = 0.032), and palmitic acid (C16:0) (*P* = 0.028) than the control group. Similarly, the total MUFAs, including palmitoleic acid (C16:1, n-7) and oleic acid (C18:1, n-9), decreased from baseline in the weight-loss group, and these decreases were significantly greater than those in the control group after adjustment for the baseline values (*P =* 0.017, *P =* 0.006, and *P =* 0.018, respectively). At follow-up, the weight-loss group showed significantly lower levels of total MUFAs (*P* = 0.021), palmitoleic acid (C16:1, n-7) (*P* = 0.018), and oleic acid (C18:1, n-9) (*P* = 0.024) than did control group. Although the total n-6 and n-3 PUFA levels did not significantly change, eicosadienoic acid (C20:2, n-6) and dihomo-γ-linolenic acid (C20:3, n-6) decreased from baseline in the weight-loss group. Both showed greater reductions in the weight-loss group than in the control group after adjusting for the baseline values (*P* < 0.001 and *P* = 0.003, respectively). At follow-up, the weight-loss group showed significantly lower levels of eicosadienoic acid (C20:2, n-6) (*P* < 0.001) and dihomo-γ-linolenic acid (C20:3, n-6) (*P* = 0.001) than the control group (Table 3).

**Table 3:** GC-MS analysis of fatty acids in the weight-maintenance (control) and weight-loss groups at baseline and 12-week follow-up

Fatty acids (Relative peak area)	Weight-maintenance group (*n* = 38)			Weight-loss group (*n* = 37)			*P* ^*a*^	*P* ^*b*^	*P* ^*c*^	*P* ^*d*^
	Baseline		Follow-up	Baseline		Follow-up				
Saturated fatty acids	7.292 ± 0.211		7.287 ± 0.215	7.166 ± 0.177		6.565 ± 0.243^*^	0.650	0.029		
Change		− 0.005 ± 0.216			− 0.601 ± 0.228				0.062	0.027
Lauric acid (C12:0)	0.033 ± 0.003		0.040 ± 0.005	0.027 ± 0.002		0.027 ± 0.003	0.063	0.032		
Change		0.007 ± 0.005			0.000 ± 0.003				0.313	0.067
Myristic acid (C14:0)	0.307 ± 0.022		0.319 ± 0.029	0.260 ± 0.017		0.241 ± 0.031	0.105	0.068		
Change		0.013 ± 0.028			− 0.019 ± 0.029				0.435	0.210
Pentadecylic acid (C15:0)	0.045 ± 0.002		0.046 ± 0.003	0.041 ± 0.002		0.046 ± 0.006	0.182	0.978		
Change		0.001 ± 0.002			0.005 ± 0.006				0.505	0.466
Palmitic acid (C16:0)	4.762 ± 0.148		4.729 ± 0.135	4.646 ± 0.118		4.333 ± 0.113^*^	0.545	0.028		
Change		− 0.033 ± 0.152			− 0.313 ± 0.120				0.155	0.033
Stearic acid (C18:0)	2.011 ± 0.055		2.014 ± 0.059	2.054 ± 0.048		1.778 ± 0.108^**^	0.557	0.058		
Change		0.004 ± 0.056			− 0.276 ± 0.100				0.016	0.021
Arachidic acid (C20:0)	0.040 ± 0.002		0.041 ± 0.002	0.041 ± 0.001		0.041 ± 0.002	0.623	0.859		
Change		0.001 ± 0.002			0.000 ± 0.003				0.808	0.978
Behenic acid (C22:0)	0.096 ± 0.005		0.098 ± 0.004	0.098 ± 0.004		0.099 ± 0.004	0.663	0.880		
Change		0.003 ± 0.004			0.001 ± 0.005				0.759	0.938
Monounsaturated fatty acids	1.472 ± 0.052		1.457 ± 0.054	1.442 ± 0.046		1.235 ± 0.078^**^	0.663	0.021		
Change		− 0.015 ± 0.050			− 0.207 ± 0.068				0.026	0.017
Palmitoleic acid (C16:1, n-7)	0.196 ± 0.011		0.197 ± 0.012	0.192 ± 0.011		0.156 ± 0.012^**^	0.777	0.018		
Change		0.001 ± 0.011			− 0.035 ± 0.009				0.013	0.006
cis-10-Heptadecenoic acid (C17:1, n-7)	0.015 ± 0.001		0.014 ± 0.001	0.014 ± 0.001		0.014 ± 0.003	0.264	0.998		
Change		0.000 ± 0.001			0.001 ± 0.003				0.675	0.709
Oleic acid (C18:1, n-9)	1.193 ± 0.041		1.176 ± 0.042	1.171 ± 0.036		0.997 ± 0.066^**^	0.693	0.024		
Change		− 0.016 ± 0.039			− 0.174 ± 0.057				0.025	0.018
Eicosenoic acid (C20:1, n-9)	0.011 ± 0.001		0.012 ± 0.001	0.010 ± 0.001		0.011 ± 0.002	0.158	0.638		
Change		0.001 ± 0.001			0.001 ± 0.001				0.889	0.947
Erucic acid (C22:1, n-9)	0.008 ± 0.001		0.007 ± 0.001	0.007 ± 0.001		0.008 ± 0.001	0.576	0.476		
Change		− 0.001 ± 0.001			0.001 ± 0.001				0.278	0.321
Nervonic acid (C24:1, n-9)	0.049 ± 0.002		0.050 ± 0.003	0.048 ± 0.002		0.049 ± 0.002	0.669	0.731		
Change		0.001 ± 0.002			0.000 ± 0.003				0.979	0.824
Polyunsaturated fatty acids (n-6)	2.965 ± 0.065		2.971 ± 0.069	2.844 ± 0.070		2.800 ± 0.067	0.208	0.080		
Change		0.006 ± 0.055			− 0.044 ± 0.071				0.577	0.212
Linoleic acid (C18:2, n-6)	2.203 ± 0.052		2.194 ± 0.054	2.097 ± 0.051		2.089 ± 0.050	0.149	0.156		
Change		− 0.009 ± 0.049			− 0.008 ± 0.052				0.992	0.449
γ-linolenic acid (C18:3, n-6)	0.038 ± 0.003		0.041 ± 0.005	0.030 ± 0.002		0.028 ± 0.002	0.065	0.033		
Change		0.003 ± 0.004			− 0.002 ± 0.002				0.222	0.220
Eicosadienoic acid (C20:2, n-6)	0.022 ± 0.001		0.023 ± 0.001	0.021 ± 0.001		0.016 ± 0.001^***^	0.519	< 0.001		
Change		0.001 ± 0.001			− 0.005 ± 0.001				< 0.001	< 0.001
Dihomo-γ-linolenic acid (C20:3, n-6)	0.122 ± 0.005		0.125 ± 0.006	0.111 ± 0.005		0.096 ± 0.006^*^	0.168	0.001		
Change		0.004 ± 0.005			− 0.015 ± 0.007				0.028	0.003
Arachidonic acid (C20:4, n-6)	0.547 ± 0.018		0.553 ± 0.020	0.550 ± 0.020		0.537 ± 0.021	0.927	0.584		
Change		0.006 ± 0.013			− 0.013 ± 0.019				0.421	0.419
Docosatetraenoic acid (C22:4, n-6)	0.033 ± 0.002		0.034 ± 0.002	0.034 ± 0.002		0.033 ± 0.002	0.826	0.670		
Change		0.001 ± 0.002			− 0.001 ± 0.002				0.490	0.505
Polyunsaturated fatty acids (n-3)	0.477 ± 0.025		0.496 ± 0.040	0.477 ± 0.028		0.469 ± 0.034	0.999	0.619		
Change		0.018 ± 0.035			− 0.008 ± 0.025				0.542	0.541
α-linolenic acid (C18:3, n-3)	0.115 ± 0.010		0.121 ± 0.009	0.099 ± 0.007		0.093 ± 0.010	0.182	0.037		
Change		0.005 ± 0.008			- 0.007 ± 0.008				0.296	0.114
Eicosapentaenoic acid (C20:5, n-3)	0.112 ± 0.008		0.115 ± 0.016	0.110 ± 0.010		0.116 ± 0.013	0.867	0.978		
Change		0.003 ± 0.015			0.006 + ±0.012				0.894	0.926
Docosapentaenoic acid (C22:5, n-3)	0.048 ± 0.003		0.050 ± 0.004	0.046 ± 0.002		0.044 ± 0.003	0.696	0.193		
Change		0.002 ± 0.004			− 0.003 ± 0.002				0.257	0.195
Docosahexaenoic acid (C22:6, n-3)	0.203 ± 0.016		0.210 ± 0.024	0.223 ± 0.019		0.217 ± 0.018	0.425	0.808		
Change		0.007 ± 0.015			− 0.005 ± 0.012				0.513	0.527
Ratio n-6/n-3	6.786 ± 0.352		7.243 ± 0.493	6.600 ± 0.346		7.519 ± 0.673	0.707	0.742		
Change		0.457 ± 0.421			0.919 ± 0.544				0.502	0.513
C16 Δ9-desaturase^*a*^	0.041 ± 0.001		0.041 ± 0.002	0.041 ± 0.002		0.036 ± 0.002^**^	0.992	0.058		
Change		0.000 ± 0.001			− 0.005 ± 0.002				0.016	0.014
C18 Δ9-desaturase^*b*^	0.593 ± 0.012		0.583 ± 0.011	0.569 ± 0.009		0.561 ± 0.010	0.111	0.144		
Change		− 0.010 ± 0.013			− 0.008 ± 0.012				0.903	0.331
Δ6-desaturase^*c*^	0.017 ± 0.002		0.019 ± 0.002	0.014 ± 0.001		0.014 ± 0.001	0.122	0.049		
Change		0.001 ± 0.002			− 0.001 ± 0.001				0.264	0.211
Δ5-desaturase^*d*^	4.725 ± 0.201		4.689 ± 0.214	5.141 ± 0.190		6.157 ± 0.336^**^	0.137	< 0.001		
Change		− 0.035 ± 0.154			1.017 ± 0.351				0.008	0.001
Elongase^*e*^	0.426 ± 0.008		0.428 ± 0.007	0.444 ± 0.006		0.406 ± 0.014^*^	0.092	0.161		
Change		0.001 ± 0.010			− 0.038 ± 0.014				0.024	0.101

Mean ± SE. ^∮^tested following logarithmic transformation. *P*^*a*^-values derived from an independent *t-*test between the groups at baseline. *P*^*b*^-values derived from an independent *t-*test between the groups at follow up. *P*^*c*^-values derived from an independent *t-*test between the groups at changed values. *P*^*d*^-values are *P*^*c*^-values adjusted with the baseline values. ^*^P < 0.05, ^**^P < 0.01, and ^***^P < 0.001 derived from a paired *t*-test. ^*a*^C16 Δ9-desaturase = palmitoleic acid/palmitic acid. ^*b*^C18 Δ9-desaturase = oleic acid/stearic acid. ^*c*^Δ6-desatuarse = γ-linolenic acid/linoleic acid. ^*d*^Δ5-desaturase = arachidonic acid/dihomo-γ-linolenic acid. ^*e*^Elongase activity = stearic acid/palmitic acid

C16 Δ9-desaturase activity significantly decreased from baseline in the weight-loss group, and this reduction was greater in the weight-loss group than in the control group after adjusting for the baseline values (*P* = 0.014). The activity of Δ5-desaturase significantly increased from baseline in the weight-loss group, and this increase was greater in the weight-loss group than in the control group after adjusting for the baseline values (*P* = 0.001). In addition, at follow-up, Δ5-desaturase activity was significantly higher in the weight-loss group than in the control group (*P <* 0.001). Elongase activity significantly decreased after the intervention (Table 3).

### Relations between changes in L4 VFA and changes in clinical/biochemical characteristics and fatty acid levels

**Fig. 1:**
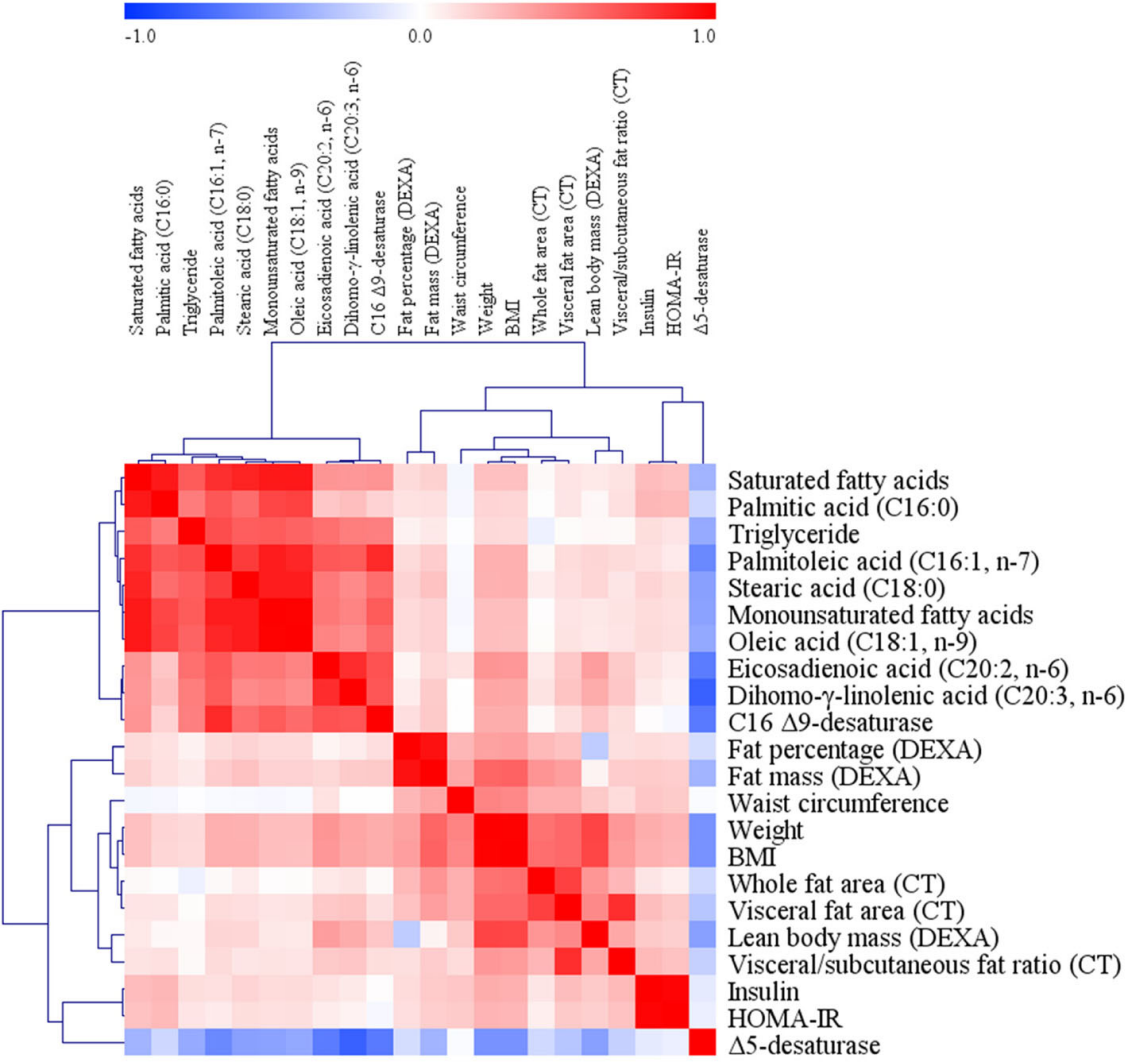
Correlation matrix among major clinical parameters, fatty acids, and fatty acid desaturases (changed values). Correlations were obtained by identifying a Pearson’s correlation coefficient. *Red* indicates a positive correlation, and *blue* indicates a negative correlation

Overall, 22 variables showed significant changes in the weight-loss group compared with the control group after adjusting for the baseline values [Table 1: weight, BMI, waist circumference, insulin, HOMA-IR, and TGs; Table 2: whole fat area, VFA, VSR, fat percentage, fat mass, and lean body mass; Table 3: SFAs, palmitic acid (C16:0), stearic acid (C18:0), MUFAs, palmitoleic acid (C16:1, n-7), oleic acid (C18:1, n-9), eicosadienoic acid (C20:2, n-6), dihomo-γ-linolenic acid (C20:3, n-6), C16 Δ9-desaturase, and Δ5-desaturase]. A correlation analysis between changes in L4 VFA and changes in these variables was performed. Of the total study subjects (*n* = 75), the changes in L4 VFA demonstrated significant positive correlations with changes in weight (*r* = 0.596, *P* < 0.001), BMI (*r* = 0.596, *P* < 0.001), waist circumference (*r* = 0.317, *P* = 0.006), insulin (*r* = 0.259, *P* = 0.025), fat percentage (*r* = 0.241, *P* = 0.037), fat mass (*r* = 0.381, *P* = 0.001), lean body mass (*r* = 0.464, *P* < 0.001), and dihomo-γ-linolenic acid (C20:3, n-6) (*r* = 0.235, *P* = 0.042) and a significant negative correlation with changes in Δ5-desaturase activities (*r* = −0.277, *P* = 0.016) (Fig. 1).

## Discussion

VFA is associated with plasma fatty acid profiles and lifestyle patterns [17]. We investigated the effects of weight loss by mild calorie restriction (a 300 kcal/day intake reduction) on circulating fatty acid profiles and desaturase activities in nondiabetic and overweight subjects with high VFA (L4 VFA ≥100 cm^2^). The 12-week intervention led to a 3.4% body weight loss and a 15% VFA loss in the participants in the weight-loss group. In addition, the study showed enhancement of plasma fatty acid profiles (SFAs and MUFAs), greater decreases in C16 Δ9-desaturase activity, and greater increases in Δ5-desaturase activity in the weight-loss group compared with the control group.

**Fig. 2:**
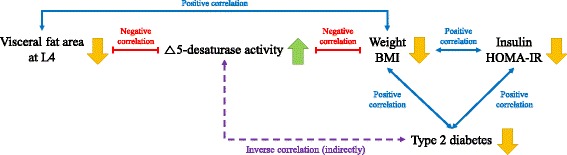
Summary of an indirect association between Δ5-desaturase activity and the risk of type 2 diabetes in the present study. Although no significant and direct correlation between Δ5-desaturase activity and the risk of type 2 diabetes was observed in this study, connections among Δ5-desaturase activity, weight, BMI, serum insulin level, and HOMA-IR score indicate an indirect inverse correlation between Δ5-desaturase activity and the risk of type 2 diabetes. Because the visceral fat area (VFA) has a significant negative correlation with Δ5-desaturase activity and Δ5-desaturase activity is inversely related to the risk of type 2 diabetes, VFA and the risk of type 2 diabetes might be positively correlated. Thus, mild calorie-restriction-induced weight loss that affects VFA at L4 decrease might lead to health benefits

An inverse relation between Δ5-desaturase activity and diabetes risk [18] and direct relations among C16 Δ9-desaturase activity, insulin resistance, and abdominal obesity [5] have been reported. In the present study, a VFA loss was significantly related to Δ5-desaturase activity but not to C16 Δ9-desaturase activity. Although some evidence supports the inverse relation between Δ5-desaturase activity and diabetes risk [18–22], the Δ5-desaturase activity of this study did not correlate to insulin and HOMA-IR, which are risk factors of type 2 diabetes. However, the weight-loss group showed greater reductions in the serum insulin levels and HOMA-IR scores than the control group; furthermore, changes in insulin and HOMA-IR were positively correlated with changes in weight and BMI, which showed a significant negative correlation with changes in Δ5-desaturase activity. Because weight and BMI are also closely related to the risk of type 2 diabetes [23, 24], our results might indicate an indirect inverse association between Δ5-desaturase activity and the risk of type 2 diabetes (Fig. 2). Consequently, mild calorie-restriction-induced weight loss in individuals with high VFA might lead to the potential benefit of a reduced risk of type 2 diabetes by improving fatty acid desaturase activity.

Estimated Δ5-desaturase activity was expressed as the ratio of arachidonic acid (20:4, n-6) to dihomo-γ-linolenic acid (20:3, n-6). The increase in Δ5-desaturase activity observed in the weight-loss group might result from the greater decrease of dihomo-γ-linolenic acid (20:3, n-6) than that observed in the control group because there were no significant changes in arachidonic acid (20:4, n-6) in the control and weight-loss groups. Indeed, changes in VFA were positively correlated with changes in dihomo-γ-linolenic acid (20:3, n-6) (Fig. 1). Therefore, the study indicated that the inverse relation between VFA and Δ5-desaturase activity might be mediated by an alteration of dihomo-γ-linolenic acid (20:3, n-6), which is also significantly associated with VFA.

Although the total n-6 PUFAs did not significantly change during the intervention, the subjects in the weight-loss group showed decreases in not only dihomo-γ-linolenic acid (C20:3, n-6) but also eicosadienoic acid (C20:2, n-6); and these reductions were greater than the reductions detected in the control group (Table 3). Consistent with our results, other studies have found that obese individuals showed higher levels of dihomo-γ-linolenic acid (C20:3, n-6) in plasma phospholipids, TGs, and sterol esters than the controls [25, 26]. A recent study emphasized the proadipogenic properties of n-6 PUFAs [27]; indeed, several studies have found that n-6 PUFAs promote adipogenesis and increase the expression of lipogenic genes [27, 28]. Muhlhausler BS et al. [27] concluded that n-6 PUFAs promote the expansion of fat depots by up-regulating both hyperplasia and hypertrophy; in other words, an accumulation of n-6 PUFAs might exacerbate an obese status by enhanced adipogenesis, which could lead to several obesity-related health risks, including IR, metabolic syndrome, and diabetes [1]. Therefore, lowering the n-6 PUFA levels by calorie-restriction-induced weight loss is necessary, and the reduction of VFA to lower n-6 PUFAs is important.

In this study, the weight-loss group showed significant decreases in total SFAs, including palmitic acid (C16:0) and stearic acid (C18:0), which have previously been observed to be related to the incidence of type 2 diabetes [29, 30]. Total MUFAs, including palmitoleic acid (C16:1, n-7) and oleic acid (C18:1, n-9), were also significantly decreased in the weight-loss group of this study. The palmitoleic acid (C16:1, n-7) plasma concentrations mostly showed de novo hepatic fatty acid synthesis from palmitic acid (C16:0) by C16 Δ9-desaturase [31, 32]; in the present study, both palmitic acid (C16:0) and C16 Δ9-desaturase activity were significantly decreased during the 12-week intervention in the weight-loss group, which indicates that these alterations might affect plasma concentrations of palmitoleic acid (C16:1, n-7). Mice supplemented with palmitoleic acid exhibit higher fat deposition, hepatic steatosis, and increased hepatic expression of sterol regulatory element-binding protein 1c and fatty acid synthase, demonstrating the pro-lipogenic effect of this MUFA [33]. Moreover, studies conducted on humans have observed a detrimental influence of this MUFA on health [34, 35]. A previous study found that high levels of palmitoleic acid (C16:1, n-7) are associated with an increased risk of cardiovascular disease [34] and are positively associated with metabolic syndrome [35], including hypertriglyceridemia [34] and abdominal obesity [36]. In the present study, the weight-loss group, which presented significantly decreased levels of palmitoleic acid (C16:1, n-7), showed greater reductions in serum TGs and waist circumference than the control group. Therefore, reduced levels of SFAs and MUFAs via calorie-restriction-induced weight and VFA loss might yield future health benefits.

One of the major points of the present study is the correlation of weight loss with a significant reduction of VFA. As discussed thus far, many changes observed in the weight-loss group of this study might be explained by the VFA change. However, there is controversy regarding whether visceral fat is more harmful to circulating fatty acids than subcutaneous fat. Jensen MD [37] reported that visceral fat is not a source of excess systemic free fatty acid availability; conversely, Björntorp P [38] concluded that visceral fat tissue could be a significant source of free fatty acids that might exert complex metabolic effects. Indeed, several studies have suggested that large visceral fat depots flooded the liver and the systemic circulation in the form of free fatty acids [39, 40]. In addition, Klein S [41] observed that excess visceral fat is more harmful than excess subcutaneous fat because the lipolysis of TGs in visceral adipose tissue releases free fatty acids into the portal vein, which directly delivers them to the liver. Nielsen et al. [42] demonstrated that the contribution of free fatty acids derived from visceral fat to the portal and systemic circulation increases with increases in the visceral fat mass. Because evidence that supports our results exists, we believe that visceral fat is an important factor affecting the circulating fatty acid profiles.

A limitation of this study is its small sample size. Additionally, our results might not be generalizable because the study subjects were limited to nondiabetic overweight Korean subjects. Finally, we did not exclude subjects who took a medication that affects lipid metabolism. Only two subjects in the control group took a lipid-lowering medication; the clinical/biochemical parameters and fatty acid profiles did not show any significant differences between the control groups according to medication [*n* = 38 (original control group) vs. *n* = 36 (control group w/o medication)] (data not shown). In addition, the baseline value-adjusted statistical significance of the changed values between the control group without medication and the weight-loss group was identical to the present results (data not shown). Therefore, in this study, medication was not considered a confounding factor in interpreting the research results. Despite these limitations, this study demonstrates that a weight-loss intervention based on a hypocaloric diet in overweight subjects with high visceral fat levels improved not only the anthropometric and biochemical parameters but also the fatty acids plasma levels.

## Conclusions

An analysis of plasma fatty acid profiles identified significant decreases in palmitic acid (C16:0), stearic acid (C18:0), palmitoleic acid (C16:1), oleic acid (C18:1), eicosadienoic acid (C20:2, n-6), and dihomo-γ-linolenic acid (C20:3, n-6) as well as a decrease in C16 Δ9-desaturase activity and an increase in Δ5-desaturase activity. These results suggest that maintenance of high VFA levels without treatment does not produce changes in the plasma fatty acid profiles, whereas even mild weight loss with reduced VFA improves the overall plasma fatty acid profile and desaturase activities. Through enhancement of the plasma fatty acid profiles, individuals may receive potential health benefits of reduced future risks of type 2 diabetes and cardiovascular disease. Moreover, eating behavior modification (mild calorie restriction) might be an effective therapy for the treatment of diseases such as type 2 diabetes and cardiovascular disease.

## Abbreviations

BMI: Body mass index
BMR: Basal metabolic rate
BP: Blood pressure
CT: Computed tomography
DEXA: Dual-energy X-ray absorptiometry
HDL: High-density lipoprotein
HOMA-IR: Homeostasis model assessment insulin resistance
IR: Insulin resistance
L4: 4th lumbar
LDL: Low-density lipoprotein
MUFA: Monounsaturated fatty acid
PUFA: Polyunsaturated fatty acid
SE: Standard error
SFA: Saturated fatty acid
TCI: Total calorie intake
TEE: Total energy expenditure
TG: Triglyceride
VFA: Visceral fat adiposity
VSR: Visceral-to-subcutaneous area ratio
